# ASSOCIATIONS BETWEEN DAILY DRINKING AND DAILY MEMORY LAPSES AMONG MIDDLE-AGED AND OLDER ADULTS IN THE MIDUS STUDY

**DOI:** 10.1093/geroni/igac059.2014

**Published:** 2022-12-20

**Authors:** Sara Miller, Jacqueline Mogle, Ashley Linden-Carmichael, David Almeida

**Affiliations:** Pennsylvania State University, University Park, Pennsylvania, United States; The Pennsylvania State University, University Park, Pennsylvania, United States; Pennsylvania State University, Pennsylvania State University, Pennsylvania, United States; The Pennsylvania State University, University Park, Pennsylvania, United States

## Abstract

Alcohol use predicts short- and long-term memory impairment. However, it is unclear how alcohol influences subjective memory, which refers to perceptions of one’s own memory functioning. This study examined associations between daily alcohol use and memory lapses (i.e., subjective memory) and evaluated perceived impact of memory lapses on daily life. Participants (n=953; Mage=55.2) were non-abstaining adults from the Midlife in the United States (MIDUS) study who participated in the 8-day daily diary project. Survey items included number of drinks and 9 memory lapse items (1=yes). After each reported memory lapse, participants indicated memory lapse irritation (range=1-10) and interference (range=1-10). Covariates included age, gender, race, education, and cognitive functioning. Multilevel logistic regression analysis revealed that within-person alcohol use (OR=1.07; p< 0.01), but not between-person alcohol use (p>0.05), was associated with greater odds of reporting any memory lapses. When assessing retrospective and prospective lapses separately, only the prediction of prospective lapses from between-person alcohol use was significant (OR=1.11; p=0.01). Finally, multilevel linear regression analysis indicated that neither within-person nor between-person alcohol use was predictive of reported irritation or interference from memory lapses (p>0.05). Study findings revealed between- and within-person associations between alcohol use and subjective memory. At the daily-level, individuals were more likely to notice memory lapses on days they drank more than usual. At the person-level, people who drank more relative to others were more likely to report prospective memory lapses. Future studies should assess how associations between alcohol use and subjective memory relate to objective cognitive functioning outcomes.

